# HPLC-MS/MS-Mediated Analysis of the Pharmacokinetics, Bioavailability, and Tissue Distribution of Schisandrol B in Rats

**DOI:** 10.1155/2021/8862291

**Published:** 2021-02-24

**Authors:** Dahu Liang, Zijing Wu, Yanhao Liu, Chao Li, Xianghong Li, Bin Yang, Haitang Xie, Hua Sun

**Affiliations:** ^1^Anhui Provincial Center for Drug Clinical Evaluation, Yijishan Hospital of Wannan Medical College, Wuhu 241001, Anhui, China; ^2^Department of Pharmacy, Bengbu First People's Hospital, Bengbu 233000, China; ^3^Wannan Medical College, Wuhu 241000, Anhui, China

## Abstract

Schisandrol B, a lignan isolated from dried *Schisandra chinensis* fruits, has been shown to exhibit hepatoprotective, cardioprotective, renoprotective, and memory-enhancing properties. This study sought to design a sensitive and efficient HPLC-MS/MS approach to measuring Schisandrol B levels in rat plasma and tissues in order to assess the pharmacokinetics, oral bioavailability, and tissue distributions of this compound *in vivo*. For this analysis, bifendate was chosen as an internal standard (IS). A liquid-liquid extraction (LLE) approach was employed for the preparation of samples that were subsequently separated with an Agilent ZORBAX Eclipse XDB-C_18_ (4.6 × 150 mm, 5 *μ*m) column with an isocratic mobile phase consisting of methanol and water containing 5 mM ammonium acetate and 0.1% formic acid (90 : 10, v/v). A linear calibration curve was obtained over the 5–2000 ng/mL and 1–1000 ng/mL ranges for plasma samples and tissue homogenates, respectively. This established method was then successfully applied to investigate the pharmacokinetics, oral bioavailability, and tissue distributions of Schisandrol B in Sprague-Dawley (SD) rats that were intravenously administered 2 mg/kg of Schisandrol B monomer, intragastrically administered Schisandrol B monomer (10 mg/kg), or intragastrically administered 6 mL/kg SCE (equivalent to 15 mg/kg Schisandrol B monomer). The oral absolute bioavailability of Schisandrol B following intragastric Schisandrol B monomer and SCE administration was approximately 18.73% and 68.12%, respectively. Tissue distribution studies revealed that Schisandrol B was distributed throughout several tested tissues, with particular accumulation in the liver and kidneys. Our data represent a valuable foundation for future studies of the pharmacologic and biological characteristics of Schisandrol B.

## 1. Introduction

*Schisandra chinensis* is a perennial deciduous woody vine that is primarily found in China, Japan, and Korea and that is a popular Traditional Chinese Medicine (TCM) that is recorded in pharmacopeias in China, Japan, and the United States [1, 2]. Recent pharmacological research suggests that *Schisandra chinensis* contains many chemical compounds including lignans, volatile oils, organic acids, vitamins, terpenoids, and polysaccharides [3]. Of these, lignans are the primary bioactive compounds therein, accounting for up to 8% of plant biomass. These lignans exhibit a range of antitumor, anti-inflammatory, antioxidant, antithrombotic, and neuroprotective activities [4–7].

Schisandrol B is a lignan that can be isolated from dried *Schisandra chinensis* fruits and that exhibits hepatoprotective, cardioprotective, renoprotective [8–12], and memory-enhancing properties [13]. There is also prior evidence that Schisandrol B can suppress or prevent vascular fibrotic disorders at least in part via disrupting TGF*β*_1_-assisted NF-*κ*B signaling [14]. In mice, Schisandrol B can also drive benign liver enlargement consistent with enhanced hepatocyte energy metabolism and energy utilization [15]. Together, these data suggest that Schisandrol B may be a pharmacologically valuable compound worthy of future research. While prior research has elucidated some of the pharmacological activities of this compound, no studies to date have comprehensively evaluated Schisandrol B monomer pharmacokinetics, bioavailability, or tissue distributions *in vivo.* As such, it is vital that a sensitive and efficient approach to measuring tissue and plasma Schisandrol B levels be developed in order to facilitate such analyses in a rat model system.

To date, several methods have been used to measure Schisandrol B levels in different sample types, including gas chromatography-mass spectrometry [16], HPLC with UV detection [17, 18], and HPLC coupled with mass spectrometry [19–24]. These approaches, however, are limited by factors including complexity, a narrow linear range, and prolonged running times. In addition, these studies have primarily evaluated Schisandrol B pharmacokinetics following the administration of complex medicinal herbal mixtures or lignan mixtures. Few studies to date have simultaneously assessed the pharmacokinetics, tissue distributions, and bioavailability of Schisandrol B *in vivo.* Herein, an HPLC-MS/MS approach was established and validated for the evaluation of these pharmacokinetic parameters in male Sprague-Daley (SD) rats administered Schisandrol B. Together, our data represent a valuable foundation for future studies of the pharmacologic and biological properties of Schisandrol B.

## 2. Material and Methods

### 2.1. Chemicals and Reagents

*Schisandra chinensis* was obtained from the Yijishan Hospital of Wannan Medical College and was positively identified by Professor Lu Jianping of Wannan Medical College (Wuhu, China). Schisandrol B (>98% pure) was obtained from Shanghai Yuanye Biological Technology Co., Ltd. Internal standard (IS) bifendate (>98% pure) was procured from Shanghai Macklin Biochemical Co., Ltd (Shanghai, China). HPLC-grade formic acid, methanol, and methyl tert-butyl ether (MTBE; Merck, Germany) were used for the present study, as was analytical grade ammonium acetate. A Milli-Q water system (Millipore, USA) was employed for the preparation of diH_2_O.

### 2.2. Apparatus

A SCIEX Triple Quad 3200 HPLC-MS/MS system (Sciex, Applied Biosystems Inc, USA) and a Shimadzu LC-20AD binary pump equipped with a SIL-HTc autosampler and CTO-10ASvp column oven (Shimadzu, Japan) were used for the present study. An Agilent ZORBAX Eclipse XDB-C_18_ (4.6 × 150 mm, 5 *μ*m) column was used for all chromatographic separation. A Pro 200 Hand-Held or Post-Mounted Laboratory Homogenizer (PRO Scientific Inc, USA) was used to prepare tissue homogenates.

### 2.3. HPLC-MS/MS Conditions

Schisandrol B chromatographic separation was carried out with a mobile phase composed of methanol with 0.1% formic acid (A) and water containing 5 mM ammonium acetate and 0.1% formic acid (90 : 10, v/v) (B). Separation was conducted at a 0.5 mL/min flow rate, while the temperature of the column and autosampler was maintained at 30°C and room temperature, respectively. A 10 *μ*L injection volume was used for these analyses. MS detection was conducted using an ESI source in positive ion mode. Ion quantification was conducted in MRM mode, with transitions of m/*z* 399.4⟶368.2 for Schisandrol B and m/*z* 387.1⟶328.3 for bifendate (IS). For these MS analyses, settings were as follows: Gas_1_ (N_2_) 40 psi; Gas_2_ (N_2_) 40 psi; ion spray voltage 5500 V; ion source temperature 550°C; curtain gas (N_2_) 25 psi. Compound-specific declustering potential (DP), collision energy (CE), entrance potential (EP), and collision cell exit potential (CXP) were 45, 29, 6, and 7 V for Schisandrol B, and 76, 28, 9, and 6 V for bifendate, respectively. Acquisition and analysis of data were evaluated with the Analyst 1.6.2 software (AB Sciex, USA).

### 2.4. Standard and Stock Solution Preparation

The preparation of stock solutions of Schisandrol B and IS was carried out by dissolving these standards in methanol at respective concentrations of 450 and 730 *μ*g/mL. These stock solutions were diluted as appropriate to prepare working solutions. Quality control (QC) samples and calibration standards were generated by adding appropriate working solution volumes to samples of blank plasma. For analyses of plasma samples, calibration standards were prepared at 5.0, 10.0, 50.0, 100.0, 500.0, 1000.0, and 2000.0 ng/mL, while the preparation of QC samples was carried out at 15 (LQC), 150 (MQC), and 1500 (HQC) ng/mL. For analyses of tissue homogenate samples, calibration standards of seven different concentrations (1.0, 5.0, 10.0, 50.0, 100.0, 500.0, and 1000.0 ng/mL) were prepared, and QC samples were prepared at 2 (LQC), 40 (MQC), and 800 (HQC) ng/mL. Additionally, the preparation of a diluted solution (500 ng/mL) of IS was carried out in methanol using the stock solution. All working solutions were kept at 4°C before use.

### 2.5. *Schisandra chinensis* Extract (SCE) Preparation

*Schisandra chinensis* was dried and crushed, and a 50 g sample of the resultant powder was subjected to ultrasonic extraction in 400 mL of 95% ethanol for 30 minutes. This extraction procedure was repeated twice, after which these extracts were filtered, pooled, evaporated in a rotary evaporator, and resuspended in 25 mL of normal saline. The resultant SCE preparation was equivalent to 2 g of crude *Schisandra chinensis* per milliliter and was stored at 4°C before use. Optimized HPLC-MS/MS conditions were used to determine that the Schisandrol B level in this prepared extract was 2.5 mg/mL.

### 2.6. Sample Preparation

Samples of rat plasma (100 *μ*L) were combined with 10 *μ*L of the IS working solution, after which samples were shaken for 2 minutes. Next, protein was precipitated via the addition of 1 mL of methyl tert-butyl ether. IS and Schisandra B were then extracted from plasma samples via shaking for 5 minutes. Samples were then spun for 5 minutes at 4,000 rpm, and supernatants were dried at 45°C under nitrogen and were then resuspended in methanol (150 *μ*L) containing formic acid (0.1%). The final solution was then shaken for 2 minutes, after which it was spun for 5 minutes at 12,000 rpm. A 10 *μ*L volume of the resultant supernatant was then injected for HPLC-MS/MS analysis.

Tissue samples were homogenized in physiological saline (1 : 3, w/v), after which 10 *μ*L of an IS solution (250 ng/mL) was combined with 100 *μ*L of each tissue homogenate sample. These homogenates were then processed and analyzed identically to plasma samples.

### 2.7. Methodological Validation

This analytical approach was validated as per the Bioanalytical Method Validation Guidance for Industry issued by the US-FDA [25] (Food and Drug Administration 2018). Key metrics used to confirm the validity of this analytical approach were specificity, selectivity, linearity, precision and accuracy, recovery, matrix effects, and stability. These analyses were conducted prior to the assessment of plasma or tissue samples.

#### 2.7.1. Sensitivity and Specificity

For selectivity and specificity analyses, blank rat plasma or tissue homogenates and samples mixed with lower limit of quantifcation (LLOQ) concentrations of Schisandrol B or IS were analyzed in parallel with samples obtained from rats following oral SCE or Schisandrol B monomer administration in order to exclude the interference of endogenous substance.

#### 2.7.2. Calibration Curves and LLOQ

Calibration curves for Schisandrol B in rat plasma (5–2000 ng/mL) and tissue homogenates (1–1000 ng/mL) were generated by plotting analyte to IS peak area ratios against expected analyte concentrations and were fitted via a weighted least-squares linear regression approach using 1/*x*^2^ as a weighting factor. LLOQ, described as the concentration of Schisandrol B with a S/N ratio >10, was evaluated based upon six replicate analyses with a percentage relative standard deviation (RSD) < 20% and a percentage relative error (RE) not exceeding ± 20%.

#### 2.7.3. Accuracy and Precision

Intra-day accuracy and precision values were assessed by evaluating six replicate QC samples prepared at HQC, MQC, LQC, and LLOQ concentrations on a given day, whereas inter-day accuracy and precision were calculated via evaluating these same replicate samples on three subsequent days. The overall accuracy of this analytical approach was determined based upon the calculated analyte concentration as a percentage of the expected nominal concentration. Precision was defined by RSD values. The acceptable criteria were an RSD <15% (LLOQ <20%) and an accuracy within 85–115% (LLOQ: 80–120%).

#### 2.7.4. Recovery and Matrix Eﬀects

Schisandrol B extraction recovery was assessed by comparing peak area values for extracted LQC, MQC, and HQC samples to the peak area of extracted plasma or tissue homogenate samples that had been spiked with the corresponding analyte. The matrix eﬀect was determined by comparing the peak areas of Schisandrol B and IS in the spiked samples after extraction and a standard sample directly dissolved with a comparable concentration of solvent.

#### 2.7.5. Stability

Schisandrol B stability was assessed in plasma and tissue homogenate samples by assessing QC samples that had been stored under a range of conditions. To evaluate analyte benchtop stability over the course of handling, QC samples were stored at approximately 25°C for 4 h before analysis. The stability of these samples in storage was evaluated via freezing them at −80°C for 20 days, while freeze-thaw stability was assessed by passing samples through three cycles of freezing and thawing at −80°C and approximately 25°C, respectively. Autosampler stability was assessed by analyzing QC samples that had been stored for 24 h in the autosampler.

### 2.8. Pharmacokinetics, Bioavailability, and Tissue Distribution Studies

Specific pathogen-free (SPF) male SD rats (180–220 g) were obtained from Qinglong Mountain Animal Breeding Farm in Jiangning District, Nanjing, Jiangsu Province, with license number: SCXK (Su) 2017–0001. The approval for this study was provided by the Animal Ethics Committee of Wannan Medical College, and these experiments were conducted in a manner consistent with the Regulations for the Administration of Affairs Concerning Experimental Animals approved by the State Council of People's Republic of China. Animals were housed in a climate-controlled facility (22 ± 2°C, 50 ± 10% relative humidity) with free food and water access, and rats were fasted with free access to water for 12 h prior to experimentation.

For pharmacokinetic analyses, SD rats were randomized into three treatment groups. Rats in Group 1 received an intravenous tail vein injection of Schisandrol B monomer (2 mg/kg). Rats in Group 2 and Group 3 were intragastrically administered a single dose of Schisandrol B monomer (10 mg/kg) or SCE (6 mL/kg; equivalent to 15 mg/kg Schisandrol B), respectively. Samples of blood (250 *μ*L) were collected from the ocular fundus vein into heparinized 2 mL tubes. For Group 1, samples were collected at 0, 0.083, 0.25, 0.5, 0.75, 1, 2, 4, 6, 8, and 12 h postinjection, while for Group 2 samples were obtained at 0, 0.25, 0.5, 1, 2, 4, 6, 8, 12, and 24 h postoral administration, and for Group 3 samples were collected at 0, 0.25, 0.5, 1, 2, 4, 6, 8, 12, 24, and 36 h postoral administration. Blood samples were spun for 5 minutes at 4500 rpm, after which plasma was collected and stored at −80°C.

For tissue distribution studies, 18 rats were randomized into six groups (*n* = 3/group). Rats were orally administered Schisandrol B monomer (10 mg/kg), after which the samples of heart, lung, kidney, liver, spleen, and brain tissues were obtained at 0.5, 1, 2, 4, 8, and 12 h postdosing. Tissue samples were washed to remove blood using normal saline, after which they were dried using filter paper, weighed, and stored at −20°C.

### 2.9. Data Processing

Plasma and tissue Schisandrol B concentrations were determined using calibration curves. Average plasma pharmacokinetic parameters including the area under the curve (AUC) for concentrations versus time and the elimination half-life (*t*_½_) were obtained using Phoenix WinNonlin (v.8.0, CERTARA, NJ, USA) with a noncompartment model. Following intravenous or oral administration, the maximum drug plasma concentration (*C*_max_) and the time to *C*_max_ (*T*_max_) were determined based upon concentration-time curves. Absolute oral bioavailability (BA) was calculated with the equation F (%) = (AUC_ig_/AUC_iv_) × (Dose_iv_/Dose_ig_) × 100%. The concentrations of Schisandrol B in tested rat tissues were expressed in *μ*g/g and were calculated with the equation *C*_t_ = *C*_s_ × *V*_s_/W, where *C*_*t*_ represented the tissue concentration (*μ*g/g), while *C*_s_, *V*_s_, and W were the concentration (ng/mL), the volume (mL), and the weight (g) of the tissue samples, respectively. Data are given as means ± SD.

## 3. Results and Discussion

### 3.1. Mass Spectrometric Detection and Chromatographic Separation

**Figure 1:**
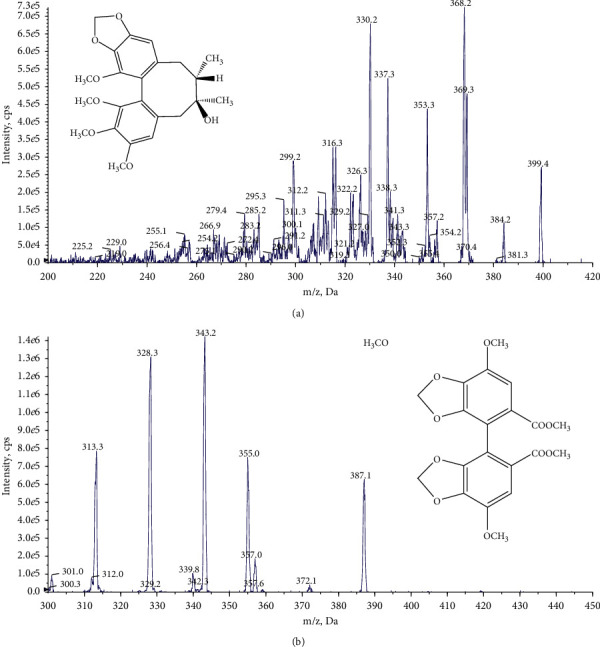
Representative multiple reaction monitoring chromatograms of Schisandrol B (a) and the internal standard bifendate (b).

To optimize ESI conditions for this study, Schisandrol B and internal standard (IS) bifendate solutions at appropriate concentrations were injected directly into the mass spectrometer. Negative as well as positive ion modes were tested in this study, and the positive ion mode was found to yield a better signal-to-noise ratio. The primary precursor molecular ion peaks of Schisandrol B and bifendate were observed at m/*z* 399.4 and 387.1, respectively. The most frequently occurring and stable product ions were observed at m/*z* 368.2 for Schisandrol B and m/*z* 328.3 for bifendate in the product ion scan. We therefore selected mass transitions of m/*z* 399.4⟶368.2 for Schisandrol B and m/*z* 387.1⟶ 328.3 for bifendate in MRM mode (Figure 1).

We initially tested both methanol-water and acetonitrile-water mobile phases to optimize chromatographic conditions. We determined that the addition of formic acid to the aqueous phase in these analyses was sufficient to improve sensitivity, while ammonium acetate improved peak shape. Methanol was found to yield a superior peak shape relative to acetonitrile and was associated with less background noise. We therefore utilized a mobile phase containing 90% methanol and 0.1% formic acid (A) and water containing 5 mmol ammonium acetate and 0.1% formic acid (90 : 10, v/v) (B). This combination yielded optimal peak shape, lower background noise, higher intensity, and shorter running times for both Schisandrol B and IS.

### 3.2. Sensitivity and Specificity

**Figure 2:**
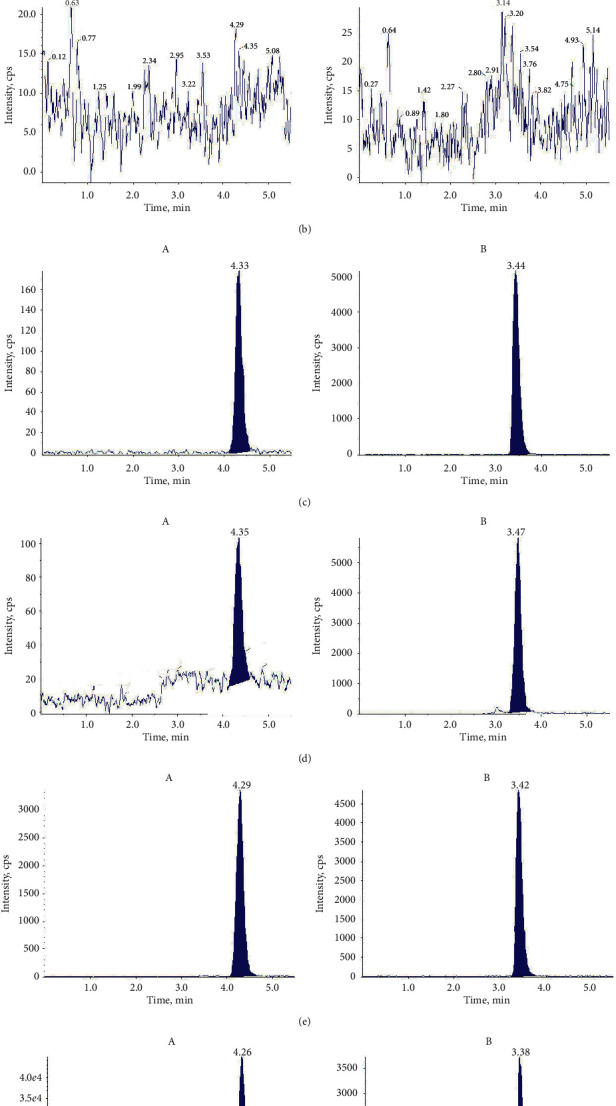
Typical chromatograms of Schisandrol B and IS in blank rat plasma (a) and liver homogenates (b); blank rat plasma (c) and liver homogenates (d) spiked with LLOQ and IS; and rat plasma (e) at 1 h and liver homogenate samples at 0.5 h (f) following the oral administration of 10 mg/kg Schisandrol B. A corresponds to Schisandrol B, and B corresponds to IS.

The specificity and selectivity of this analytical approach for IS and Schisandrol B were next assessed. Representative MRM chromatograms of pooled blank rat plasma and liver homogenate samples, as well as chromatograms from plasma and liver homogenate samples spiked with LLOQ Schisandrol B levels, plasma samples obtained at 1 h postdosing, and liver homogenate samples collected at 0.5 h postdosing, are shown in Figure 2.

No endogenous substances were found to interfere with Schisandrol B or IS retention time owing to the elevated level of MRM selectivity.

### 3.3. Linearity and LLOQ

Calibration curves for homogenates (plasma and tissue) were found to exhibit significant linearity in the 5–2000 ng/mL and 1–1000 ng/mL ranges, respectively. The LLOQ for Schisandrol B in rat plasma and tissue homogenates was 5 ng/mL and 1 ng/mL, respectively, as depicted in Table 1. These values were sufficient to facilitate pharmacokinetic analyses and tissue distribution studies.

### 3.4. Precision and Accuracy

The precision and accuracy of this analytical approach were next tested at four QC levels (LLQC, LQC, MQC, HQC), as shown in Table 2. For plasma and tissue homogenate samples, the intra- and interday precision (RSD) were not >15%, and the accuracy (RE) was within the ±15% range. All of these results were consistent with the acceptance criteria for the present study, indicating that this analytical approach was a reproducible and accurate means of measuring Schisandrol B levels in rat plasma and tissue homogenates.

### 3.5. Matrix Effect and Extraction Recovery

Extraction recovery values at all three tested concentration levels were >75%, indicating that this extraction approach was reliable and efficient. The matrix effect of Schisandrol B in plasma and tissue homogenate samples ranged within 90.62%–114.08%, suggesting that matrix did not significantly interfere with Schisandrol B analyses (Table 3).

### 3.6. Stability

Stability was evaluated by assessing analyte levels in six replicate low and high concentration QC samples stored under a range of conditions. No significant differences in Schisandrol B concentrations were observed under any of these tested conditions for either QC sample type (Table 4), suggesting that Schisandrol B is relatively stable in both samples under relevant processing and storage conditions.

### 3.7. Pharmacokinetic Study

**Figure 3:**
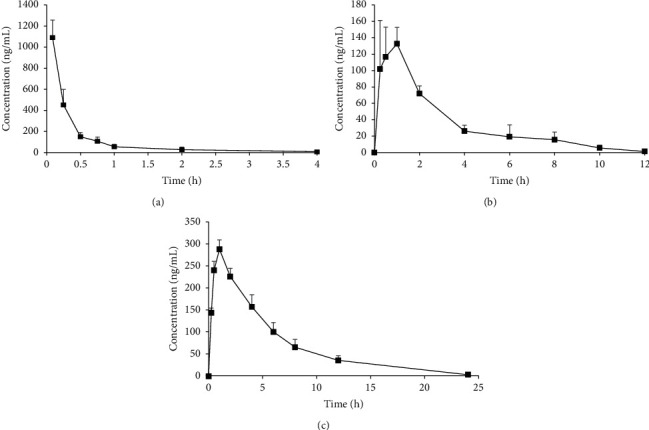
Mean plasma concentration-time curves for Schisandrol B following the intravenous administration of 2 mg/kg of monomer (a), the intragastric administration of 10 mg/kg monomer (b), and the intragastric administration of 6 mL/kg SCE (equivalent to 15 mg/kg of Schisandrol B monomer) (c).

After successfully validating this method, we assessed the pharmacokinetics of Schisandrol B in rats following intravenous Schisandrol B monomer administration (2 mg/kg), intragastric Schisandrol B monomer administration (10 mg/kg), or intragastric SCE administration (6 mL/kg; equivalent to 15 mg/kg Schisandrol B monomer). Schisandrol B mean plasma concentration-time profiles and key pharmacokinetic parameters were calculated based on these analyses (Figure 3, Table 5). In these three experimental groups, AUC_0-*t*_ (ng.h/mL) values were 456.68 ± 91.24, 426.22 ± 32.64, and 2288.87 ± 276.44 respectively, while AUC_0-∞_(ng.h/mL) values were 456.84 ± 91.63, 427.93 ± 33.15, and 2334.08 ± 381.07, respectively. Schisandrol B monomer was found to exhibit an oral bioavailability of 18.73%, whereas the bioavailability of Schisandrol B in SCE was as high as 68.12%. When comparing the pharmacokinetics of Schisandrol B in rats administered SCE to those in rats administered Schisandrol B monomer, we found that Schisandrol B derived from SCE exhibited a longer *T*_1/2_ (2.55 ± 0.66 vs. 1.59 ± 1.42), a longer MRT_0-∞_ (9.09 ± 1.51 vs. 3.31 ± 0.46), a lower CL (6.43 ± 1.83 vs. 23.37 ± 3.25), a later *T*_max_ (2.00 ± 0.87 vs. 0.54 ± 0.40), and a higher dosage exposure and bioavailability (68.12% vs. 18.73%) relative to Schisandrol B monomer at an equivalent dosage. Previous data indicated that Schisandrol B monomer is eliminated within 12 h of absorption, whereas this period extends for 24 h following SCE administration. As such, the final collection time points in Groups 2 and 3 were 24 h and 36 h, respectively. This suggests that other components in SCE may enhance the absorption of Schisandrol B, decreasing its elimination and enhancing its bioavailability.

### 3.8. Tissue Distribution Study

**Figure 4:**
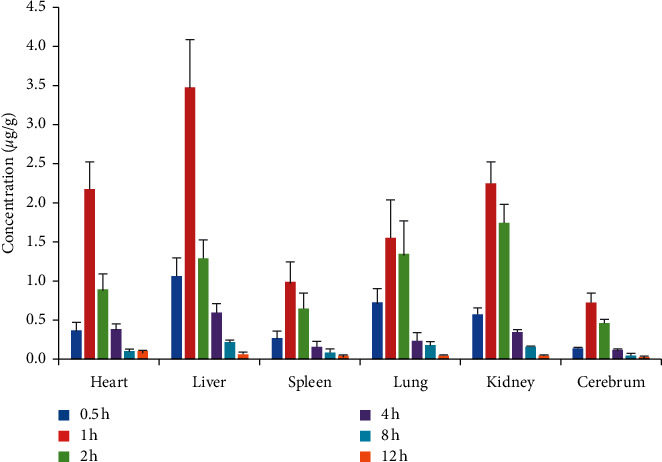
The concentration-time profile of Schisandrol B in tissues following oral administration (*n* = 3).

Having successfully validated this HPLC-MS/MS approach, it was additionally used to assess Schisandrol B concentrations in heart, liver, spleen, lung, kidney, and brain homogenates prepared at 0.5, 1, 2, 4, 8, and 12 h after the single dose (10 mg/kg) intragastric administration of Schisandrol B. The tissue distribution profiles of Schisandrol B were assessed over time (Figure 4), revealing that this compound was rapidly absorbed and readily diffused throughout all analyzed tissues. Following its oral administration, maximal Schisandrol B concentrations were observed in all analyzed tissues at 1 hour postdosing, after which they declined markedly over the following 12 hours, indicating that Schisandrol B does not accumulate substantially in any of the analyzed tissues. Compared to plasma levels in rat which were intragastrically administered a single dose of Schisandrol B monomer (10 mg/kg), the *T*_max_ values for these tissues were ∼0.5 hours slower, confirming that the absorption of oral Schisandrol B monomer requires processing such that some amount of time is required for Schisandrol B to affect target organs. Maximal Schisandrol B concentrations were detected in the liver, followed by the kidneys, heart, lung, spleen, and brain, respectively. This suggests that Schisandrol B may be best-suited to the treatment of hepatic diseases. Given their high levels of vascularization and blood flow, it is perhaps unsurprising that the liver and kidneys exhibited the highest levels of Schisandrol B, which is likely to be primarily metabolized in the former and excreted via the latter of these organs. Given that we were also able to detect Schisandrol B in the brain following its oral administration, this suggests that it can penetrate the blood-brain barrier and may thus warrant future study as a potentially valuable pharmaceutical agent capable of targeting the central nervous system.

## 4. Conclusion

Herein, we developed and validated a rapid and sensitive HPLC-MS/MS-based approach to evaluating Schisandrol B levels in rat plasma and tissue homogenates. This study is the first time to our knowledge to have assessed Schisandrol B oral bioavailability and tissue distribution patterns. We determined that the absolute oral bioavailability of Schisandrol B monomer was 18.73%, while for SCE it was 68.12%. We also found that Schisandrol B preferentially localized to the liver, kidneys, heart, and brain, providing a scientific basis for the beneficial effects of this compound on these organs. Together, our data offer a foundation for future studies of the pharmacological activity of Schisandrol B *in vivo*.

## Figures and Tables

**Table 1:** Summary of LLOQ, linear ranges, regression equations, and correlation coefficients of analytes in rat plasma and tissue homogenate samples.

Sample	Calibration curve	*R* ^2^	Linear range (ng/mL)
Plasma	*y* = 0.0081*x* − 0.0137	0.990	5–2000
Heart	*y* = 0.00825*x* − 0.00473	0.997	1–1000
Liver	*y* = 0.00868*x* − 0.00608	0.990	1–1000
Spleen	*y* = 0.0292*x* + 0.00648	0.995	1–1000
Lung	*y* = 0.00127*x* + 0.000556	0.993	1–1000
Kidney	*y* = 0.00443*x* + 0.00519	0.990	1–1000
Brain	*y* = 0.00445*x* + 0.00258	0.997	1–1000

**Table 2:** Precision and accuracy of the approach to Schisandrol B measurement in biological samples (*n* = 6).

Samples	Spiked conc (ng/mL)	Intra-day (*n* = 6)			Inter-day (*n* = 6 × 3)		
		Measured conc. (ng/mL)	Precision (RSD, %)	Accuracy (RE, %)	Measured conc. (ng/mL)	Precision (RSD, %)	Accuracy (RE, %)
Plasma	5	5.46 ± 0.26	4.73	9.22	5.45 ± 0.27	4.99	8.99
	15	13.60 ± 1.40	10.32	−9.31	13.68 ± 1.43	10.46	−8.78
	150	145.30 ± 13.05	8.98	−3.14	143.58 ± 14.11	9.82	−4.28
	1500	1436.42 ± 93.55	6.51	−1.08	1410.53 ± 112.67	7.99	−5.96

Heart	1	0.99 ± 0.08	7.91	−1.27	0.97 ± 0.03	3.08	−3.41
	2	2.00 ± 0.22	10.82	0.17	2.01 ± 0.02	0.96	0.50
	40	36.90 ± 0.39	1.06	−7.74	37.11 ± 0.22	0.59	−7.22
	800	789.33 ± 48.32	6.12	−1.33	753.10 ± 32.12	4.27	−5.86

Liver	1	0.98 ± 0.02	2.47	−2.16	0.96 ± 0.06	6.50	−4.06
	2	1.83 ± 0.06	3.11	−8.49	1.85 ± 0.02	0.90	−7.53
	40	38.32 ± 0.96	2.51	−4.20	38.09 ± 0.21	0.55	−4.78
	800	713.49 ± 7.44	1.04	−10.81	735.12 ± 18.94	2.58	−8.11

Spleen	1	0.96 ± 0.03	3.24	−4.17	0.95 ± 0.03	2.66	−4.52
	2	1.90 ± 0.10	5.02	−5.17	1.85 ± 0.05	2.80	−7.31
	40	40.18 ± 1.77	4.42	0.45	39.38 ± 0.78	1.97	−1.54
	800	761.79 ± 53.22	6.99	−4.48	762.48 ± 9.57	1.26	−4.69

Lung	1	1.00 ± 0.10	10.02	0.10	1.01 ± 0.02	1.52	0.72
	2	2.06 ± 0.10	4.82	2.82	2.03 ± 0.06	3.12	1.41
	40	36.27 ± 2.27	6.26	−9.32	37.01 ± 0.88	2.37	−7.47
	800	722.25 ± 43.09	5.97	9.72	723.66 ± 4.49	0.62	−9.54

Kidney	1	0.90 ± 0.05	6.08	−10.33	0.97 ± 0.07	6.96	−2.93
	2	1.85 ± 0.11	6.06	−7.70	1.88 ± 0.04	2.08	−5.86
	40	41.92 ± 3.88	9.26	4.81	40.94 ± 0.88	2.15	2.36
	800	721.96 ± 30.57	4.23	9.76	748.65 ± 25.22	3.37	−6.42

Brain	1	1.03 ± 0.06	6.25	2.73	0.98 ± 0.05	5.10	−2.02
	2	2.04 ± 0.06	3.07	1.95	1.89 ± 0.14	7.31	−5.47
	40	43.12 ± 2.37	5.51	7.81	39.06 ± 3.55	9.09	−3.35
	800	749.13 ± 31.01	4.14	−6.36	736.89 ± 13.01	1.77	−7.89

**Table 3:** Recovery and matrix effects of Schisandrol B in plasma and tissues homogenate samples (*n* = 6).

Samples	Conc. (ng/mL)	Extraction recovery (mean ± SD)	Matric effect (mean ± SD)
Plasma	15	75.55 ± 11.85	103.68 ± 9.77
	150	87.72 ± 10.91	110.32 ± 13.18
	1500	87.44 ± 11.36	105.71 ± 5.72

Heart	2	78.9 ± 9.10	101.31 ± 8.79
	40	76.7 ± 4.40	99.88 ± 3.09
	800	75.5 ± 10.70	101.38 ± 14.30

Liver	2	97.89 ± 6.19	106.57 ± 12.41
	40	96.03 ± 10.05	114.08 ± 4.92
	800	87.79 ± 10.74	107.39 ± 7.02

Spleen	2	83.33 ± 8.40	94.56 ± 12.11
	40	91.12 ± 5.47	101.61 ± 9.46
	800	79.99 ± 3.04	105.42 ± 2.18

Lung	2	93.19 ± 5.16	107.50 ± 4.81
	40	96.52 ± 6.81	95.92 ± 12.51
	800	92.41 ± 12.10	99.31 ± 8.43

Kidney	2	86.40 ± 9.95	113.13 ± 3.42
	40	91.03 ± 6.55	101.98 ± 3.54
	800	86.45 ± 5.00	105.82 ± 1.66

Brain	2	91.30 ± 3.29	90.62 ± 7.57
	40	94.26 ± 1.79	93.93 ± 9.74
	800	82.60 ± 9.80	96.85 ± 13.33

**Table 4:** Stability of Schisandrol B in rat plasma and tissues homogenate samples under various conditions (*n* = 6).

Samples	Spiked conc. (ng/mL)	Measured conc.			
		Bench-top (for 4 h)	Autosampler (at room temperature for 24 h)	Three freeze-thaw cycles (at −80°C)	Long term (at −80°C for 20 days)
Plasma	15	13.95 ± 1.21	14.04 ± 1.21	13.70 ± 0.98	13.92 ± 0.96
	1500	1345.92 ± 97.42	1433.13 ± 111.16	1333.92 ± 58.09	1387.98 ± 72.00

Heart	2	2.00 ± 0.25	2.03 ± 0.13	1.80 ± 0.09	1.91 ± 0.16
	800	728.09 ± 12.10	741.90 ± 66.63	743.67 ± 44.47	732.25 ± 32.14

Liver	2	1.86 ± 0.05	1.86 ± 0.04	1.81 ± 0.06	1.82 ± 0.21
	800	748.70 ± 10.92	743.18 ± 18.64	761.94 ± 36.57	756.91 ± 42.36

Spleen	2	1.80 ± 0.01	1.87 ± 0.07	1.85 ± 0.11	1.84 ± 0.09
	800	753.27 ± 8.88	772.37 ± 10.61	781.57 ± 22.05	764.19 ± 25.38

Lung	2	1.96 ± 0.24	2.07 ± 0.16	1.89 ± 0.07	1.93 ± 1.98
	800	728.69 ± 21.07	720.05 ± 15.93	777.29 ± 42.83	748.07 ± 38.92

Kidney	2	1.92 ± 0.25	1.88 ± 0.11	1.88 ± 0.04	1.86 ± 0.17
	800	772.08 ± 88.79	751.92 ± 50.02	773.92 ± 47.36	766.91 ± 50.09

Brain	2	1.77 ± 0.19	1.87 ± 0.12	1.98 ± 0.04	1.80 ± 0.11
	800	723.23 ± 57.23	738.33 ± 8.57	732.87 ± 49.33	729.24 ± 37.43

**Table 5:** Pharmacokinetic parameters of Schisandrol B in rat plasma.

Parameters	Schisandrol B		SCE
	Group 1 (2 mg/kg; iv)	Group 2 (10 mg/kg; ig)	Group 3 (6 mL/kg; ig)
*t*_1/2_ (h)	1.10 ± 0.10	1.59 ± 1.42	2.55 ± 0.66
CL (L/h/kg)	4.38 ± 1.12	23.37 ± 3.25	6.43 ± 1.83
MRT_0-∞_ (h)	0.67 ± 0.01	3.31 ± 0.46	9.09 ± 1.51
*T*_max_ (h)	—	0.54 ± 0.40	2.00 ± 0.87
*C*_max_ (ng/mL)	—	133.09 ± 18.71	288.67 ± 67.47
AUC_0-t_ (ng.h/mL)	456.68 ± 91.24	426.22 ± 32.64	2288.87 ± 276.44
AUC_0-∞_ (ng.h/mL)	456.84 ± 91.63	427.93 ± 33.15	2334.08 ± 381.07
F (%)	—	18.73	68.12

## Data Availability

The data used to support the findings of this study are available from the corresponding author upon request.
